# Artificial intelligence-based classification of bone tumors in the proximal femur on plain radiographs: System development and validation

**DOI:** 10.1371/journal.pone.0264140

**Published:** 2022-02-24

**Authors:** Chan-Woo Park, Seong-Je Oh, Kyung-Su Kim, Min-Chang Jang, Il Su Kim, Young-Keun Lee, Myung Jin Chung, Baek Hwan Cho, Sung-Wook Seo

**Affiliations:** 1 Department of Orthopedic Surgery, Samsung Medical Center, Sungkyunkwan University School of Medicine, Seoul, Korea; 2 Medical AI Research Center, Samsung Medical Center, Seoul, Korea; 3 Department of Health Sciences and Technology, SAIHST, Sungkyunkwan University, Seoul, Korea; 4 Department of Radiology, Samsung Medical Center, Sungkyunkwan University School of Medicine, Seoul, Korea; 5 Department of Medical Device Management and Research, SAIHST, Sungkyunkwan University, Seoul, Korea; Universiti Malaysia Pahang, MALAYSIA

## Abstract

**Purpose:**

Early detection and classification of bone tumors in the proximal femur is crucial for their successful treatment. This study aimed to develop an artificial intelligence (AI) model to classify bone tumors in the proximal femur on plain radiographs.

**Methods:**

Standard anteroposterior hip radiographs were obtained from a single tertiary referral center. A total of 538 femoral images were set for the AI model training, including 94 with malignant, 120 with benign, and 324 without tumors. The image data were pre-processed to be optimized for training of the deep learning model. The state-of-the-art convolutional neural network (CNN) algorithms were applied to pre-processed images to perform three-label classification (benign, malignant, or no tumor) on each femur. The performance of the CNN model was verified using fivefold cross-validation and was compared against that of four human doctors.

**Results:**

The area under the receiver operating characteristic (AUROC) of the best performing CNN model for the three-label classification was 0.953 (95% confidence interval, 0.926–0.980). The diagnostic accuracy of the model (0.853) was significantly higher than that of the four doctors (0.794) (P = 0.001) and also that of each doctor individually (0.811, 0.796, 0.757, and 0.814, respectively) (P<0.05). The mean sensitivity, specificity, precision, and F1 score of the CNN models were 0.822, 0.912, 0.829, and 0.822, respectively, whereas the mean values of four doctors were 0.751, 0.889, 0.762, and 0.797, respectively.

**Conclusions:**

The AI-based model demonstrated high performance in classifying the presence of bone tumors in the proximal femur on plain radiographs. Our findings suggest that AI-based technology can potentially reduce the misdiagnosis of doctors who are not specialists in musculoskeletal oncology.

## Introduction

The proximal part of the femur, i.e., the head, neck, and trochanteric areas, is one of the most common anatomic locations for benign bone tumors and tumor-like conditions [1] and a common location for bone metastasis of malignant tumors from other organs. Primary malignancies, such as osteosarcoma, chondrosarcoma, and Ewing’s sarcoma can also develop at the proximal femur [2–4]. As high mechanical stress is concentrated during weight-bearing activities, it is also the most common site of pathological fractures secondary to bone tumors [5, 6]. Therefore, special attention should be paid to tumors involving the proximal femur.

Early detection and classification should be performed to ensure successful treatment of bone tumors. For primary malignancies, radical surgical resection is possible only when they are detected in the early stages [2, 3]. Protected weight-bearing, surgical augmentation, or radiotherapy may be needed to prevent fractures around osteolytic tumors [5, 6]. Although plain radiographs are widely used for routine screening for bone tumors, a considerable rate of misdiagnosis upon visual examination has been reported, as bone tumors show various morphologies and common ambiguous features [4, 7, 8]. Computed tomography (CT), magnetic resonance imaging (MRI), bone scan, and positron emission tomography (PET) are more sensitive in detecting bone tumors; however, the routine use of advanced imaging modalities is costly and time-consuming.

Advancements in artificial intelligence (AI) technologies are bringing innovations in medical data analysis [8, 9]. Deep learning, a high-level neural network resembling the human brain, solves complex problems that low-level artificial intelligence cannot. Of these, convolutional neural network (CNN) models have shown high performance in analysing medical images with complex patterns. The ability of deep learning in interpreting two-dimensional medical images has become similar to that of an average human expert in the field [10]. Various studies have reported excellent results in diagnosing or classifying a disease using plain radiography, ultrasound, CT, MRI, microscopy, and endoscopy [9, 11–13]. In this regard, AI technology may potentially be used to detect bone tumors on plain radiographs. If the AI-based classification system performs well in clinical practice, the time, cost, and human errors can be dramatically reduced.

This study aimed to develop and validate an AI classifier to diagnose bone tumors in the proximal femur on plain hip radiographs. We pre-processed and optimized hip images so that the deep learning model could achieve higher performance in recognizing lesions in the proximal femur. Various CNN algorithms have been used to detect and classify bone tumors, and their performances have been evaluated against practicing orthopaedic surgeons with varying experience levels.

## Materials and methods

### Data collection and labelling

This study was conducted with the approval of the institutional review board. We collected 269 standard hip radiographs showing both proximal femurs in the format of digital imaging and communications in medicine [14]. All radiographs were taken between January 2008 and December 2019 at a single center. The presence of benign or malignant tumors in the proximal femur was confirmed using MRI and tissue specimens. Of the 269 hip radiographs, 89 were malignant, 120 were benign, and 60 had no tumors in the proximal femur. Images with any fracture or surgical fixation were excluded from the study to avoid misunderstanding the deep learning models.

### Image pre-processing and augmentation

**Fig 1:**
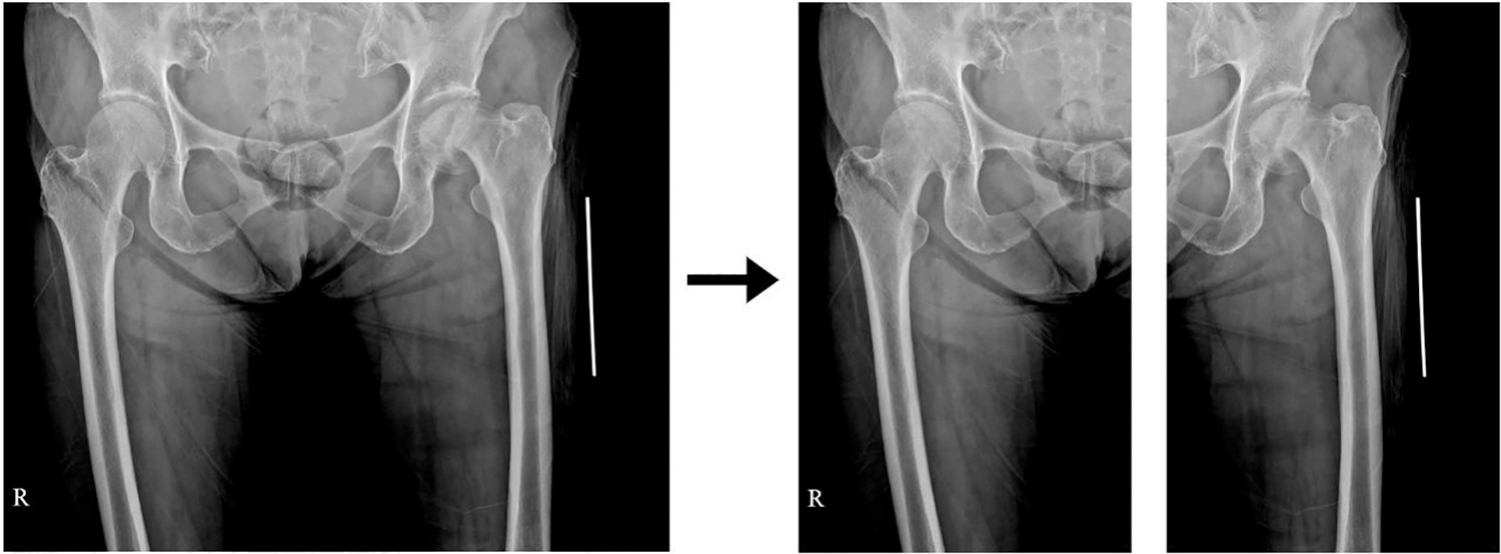
Example of an original hip radiograph presented asymmetrically on the horizontal axis. As the center of the body was not positioned at the exact center of the radiographic image, this caused the image to be skewed to one side when it was cut in half.

The hip radiographs were normalised using the min-max normalisation technique [15]. This technique uses a number between 0 and 1 to map the minimum and maximum pixel values of each image where the amount of radiation exposure and brightness slightly differ. As only five hip radiographs had tumors on both sides of the femur, the amount of training data was insufficient to determine such cases. Therefore, we introduced an automatic technique that divides the image vertically in half and flips the right-side image to the left. However, the pubic symphysis of most patients was not positioned at the exact center of the radiographic image. As shown in Fig 1, this caused the image to be skewed to one side when the image was cut in half. To find the proper center of division in asymmetrical images, we applied the following four pre-processing stages.

**Fig 2:**
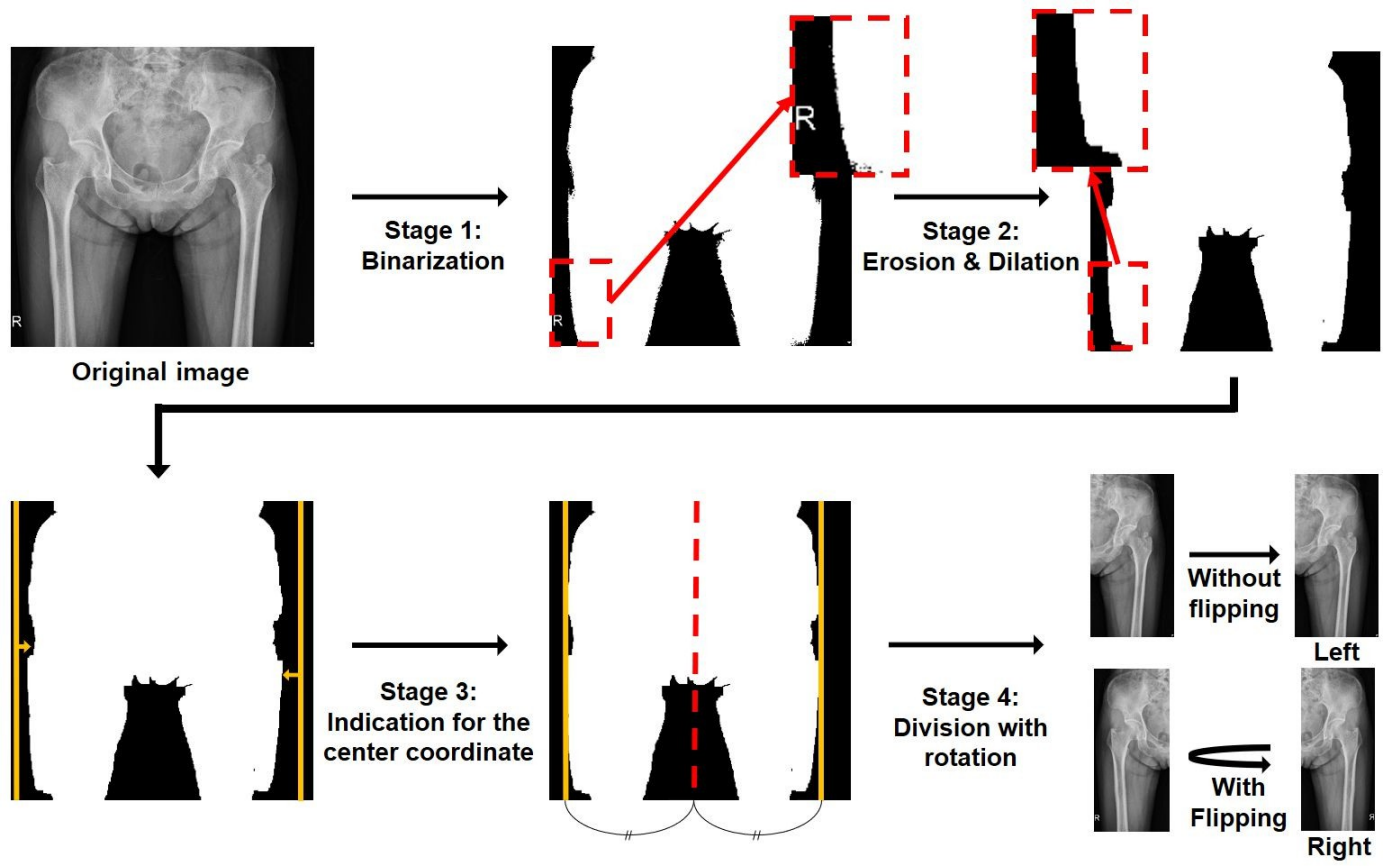
Multiple pre-processing stages for a radiographic image. Stage 1: Binarization process giving a full pixel value to every non-zero pixel. Stage 2: Erosion and dilation process to remove artifacts in the image. Stage 3: Calculation of the center coordinate (red line) to divide and flip the image. Stage 4: Division and flipping process of femurs to be aligned with the same (i.e., left) side.

First, the images were binarized by assigning a full pixel value (i.e., 1) to every non-zero pixel (Fig 2) (stage 1). Subsequently, the erosion and dilation processes were introduced to remove artifacts, such as the “R”-shaped marker (stage 2) [16]. The right and left end coordinates of the remaining body area were identified (stage 3), and the center line was set between these two coordinates. Finally, the images were divided in half by the center line, flipped (all right images to the left), and saved in PNG format (stage 4). Through these four stages, a total of 538 femoral images were aligned with the same (i.e., left) side, thereby making the shape of the femurs more homogenous for the input data. The number of images for patients with malignant tumors, benign tumors, and those without tumors increased from 89, 120, and 60 to 94, 120, and 324, respectively.

**Fig 3:**
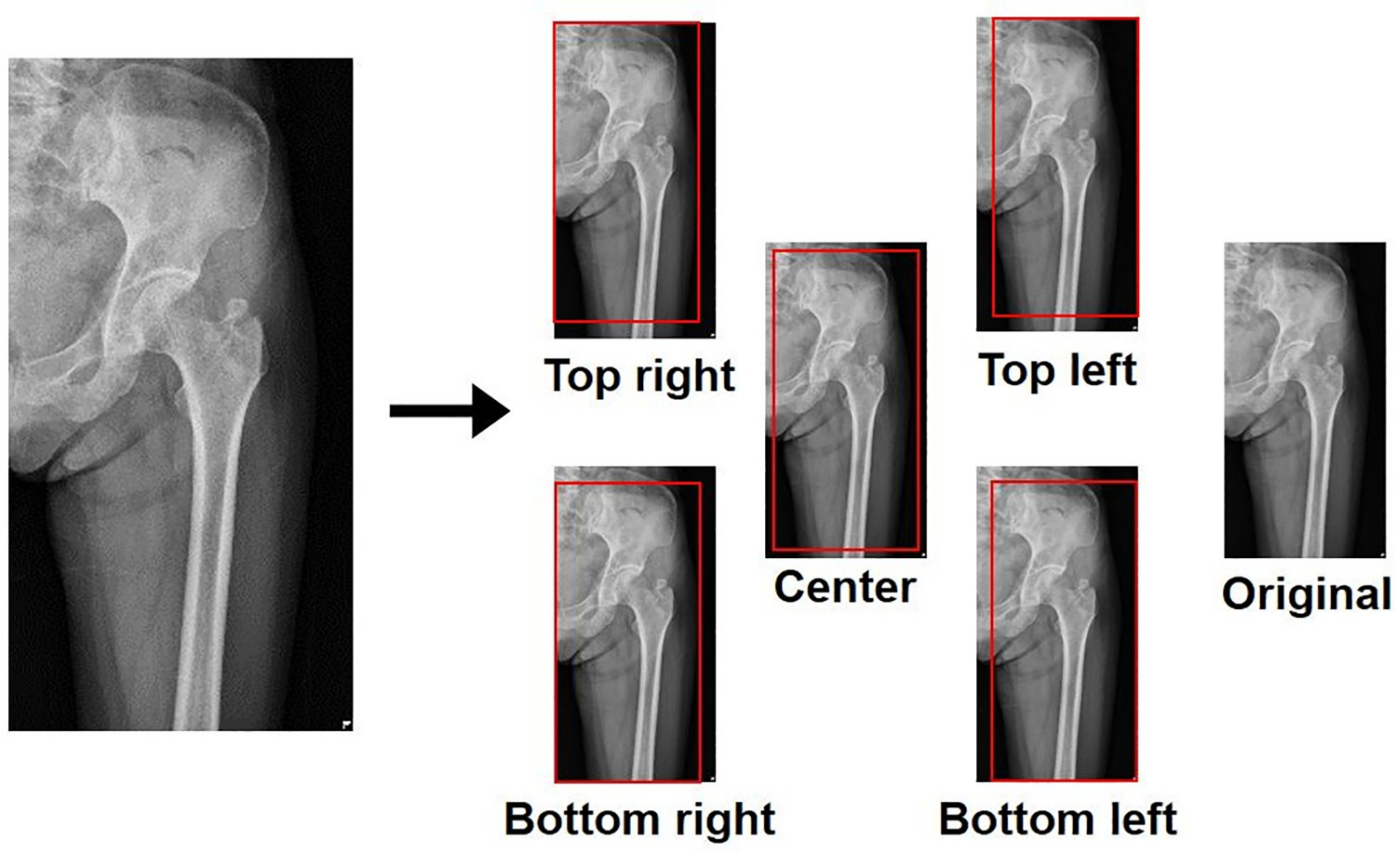
Illustrations showing the cropping process. Five specific fields were selected to be located in the top right, bottom right, bottom left, top left, and center of the images. The images were cut in these fields to be 10% smaller than their original size, and were rescaled to their original size.

The cropping process was applied for data augmentation, as shown in Fig 3. Five specific fields were selected to be located in the top right, bottom right, bottom left, top left, and center of the images. Then, the images were cut in these fields to be 10% smaller and rescaled to their original size. By this process, the total number of training images could be increased six times [17]. The overall pre-processing was finally completed by performing equalization [18] and image resizing. We applied equalization [18] to balance the overall image brightness and darkness, and resized their height and width to 500 and 300, respectively, as the aspect ratio of the original images was approximately 1.7:1.

### Application of CNN models

Several CNN algorithms were applied to the classification system, including ResNet 50 [19]; GoogleNet Inception v3 [20]; and EfficientNet-b1, b2, and b3 [21], all of which performed well in various real-world image classification tasks. For better optimization of these models, we applied a transfer learning technique that used the weight of each model pre-trained with the ImageNet dataset as an initial network parameter [22].

**Fig 4:**
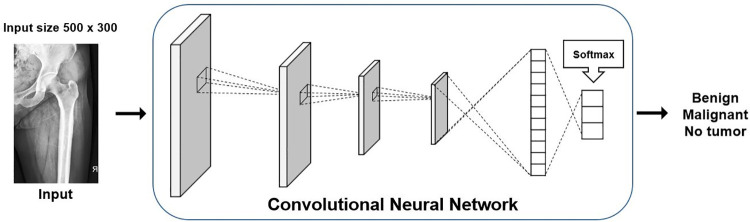
Deep learning framework for classification of bone tumors in the proximal femur. The convolutional neural network model was trained to classify each image into one of the 3 classes (benign, malignant, and no tumor).

We trained each CNN model to perform three-label classifications (benign, malignant, or no tumor) on each femur, as illustrated in Fig 4. We also set the number of input images differently according to the input requirements of each CNN model. In the case of ResNet50 and Inception v3, target images were duplicated thrice, as these models require three input channels. Because EfficientNet models can be configured as one input channel, each image was used as an input without such duplication. Except for the number of input channels, we used the same number of epochs, input image size, learning optimizer, learning rate per each epoch, and loss function when training and testing different models. The settings were finally adjusted to produce the highest classification performance of the best performing model. Epoch was set to 50, and the batch size was set to 8. Learning rates started from 0.1, divided by 10 every 10 epochs, and stochastic gradient descent was used as the optimizer. Cross entropy was used as the loss function for model training and validation. We implemented the system on PyTorch with a single graphic-processing unit of NVIDIA GeForce GTX 1080Ti.

### Performance evaluation

The performance of each CNN model for three-label classification was evaluated using the following six measurements: the area under curve (AUC) for receiver operator characteristics (ROC), accuracy, sensitivity, specificity, precision, and F1 score. Accuracy refers to the proportion of the total number of test samples in which the CNN identifies the true labels. Precision and sensitivity are defined as the class-wise averages of proportions correctly detected among all samples detected by target class and all samples of the target class, respectively. The F1 score denotes the harmonic mean of precision and sensitivity. As the task was three-label classification, we calculated three groups, namely, true positive (TP), false positive (FP), and false negative (FN), by selecting a target label *i* ∈ {1, 2, 3} as positive and other labels, excluding the target label, as negative.

$$Accuracy=\frac{\sum_{i=1}^{3}T_{i}}{D_{test}}$$

$$Precision=\frac{1}{3}*\sum_{i=1}^{3}{Precision}_{i}=\frac{1}{3}*\sum_{i=1}^{3}\frac{{TP}_{i}}{{TP}_{i}+{FP}_{i}}$$

$$Sensitivity=\frac{1}{3}*\sum_{i=1}^{3}{Sensitivity}_{i}=\frac{1}{3}*\sum_{i=1}^{3}\frac{{TP}_{i}}{{TP}_{i}+{FN}_{i}}$$

$$F1\_score=\frac{1}{3}*\sum_{i=1}^{3}{F1\_score}_{i}=\frac{1}{3}*\sum_{i=1}^{3}2*\frac{{Precision}_{i}\times {Sensitivity}_{i}}{{Precision}_{i}+{Sensitivity}_{i}}$$

where *T_i_* is the number of testing samples with both label and estimate equal to *i*. *D_test_* is the total number of testing samples, and *TP_i_, FP_i_*, and *FN_i_* indicate true positive, false positive, and false negative, respectively. *Precision_i_, Sensitivity_i_*, and *F*1 *score_i_* indicate the precision, sensitivity, and F1 score, respectively, when a label *i* is selected as positive. The final values of precision and sensitivity were calculated using class-wise averages of each value.

### Examination by human doctors

In order to better understand the clinical efficacy of the deep learning models, their performance was compared to those of the human doctors. Four orthopaedic surgeons participated in the examination of the same hip radiographs as those used for training of the CNN models. The doctors were composed of two general orthopaedic surgeons and two musculoskeletal tumor specialists. To ensure the fairness of the test, doctors who had participated in the ground truth labelling of the corresponding data were excluded. As human doctors usually find lesions by comparing two femurs on a single radiograph, the original hip radiographs showing both femurs were provided. All clinical information, including age, sex, name, patient number, and date, was blinded before the examination. The doctors were asked to perform the three-label classification on each of the left and right femurs.

### Statistical analysis

The accuracy, sensitivity, specificity, precision, and F1 score for three-label classification of each CNN model and human doctor were evaluated by fivefold cross-validation [23]. These values were described as the mean and standard deviation. The AUC for each CNN model was calculated according to the micro-average scale (i.e., the average AUC of each class), and 95% confidence intervals (CIs) were described. The diagnostic accuracy for three-label classification was compared between the best-performing CNN model and each of the human doctors using the chi-squared test. All statistical analyses were performed using SPSS Statistics version 27.0 software (IBM Corp., Armonk, NY, USA). Statistical significance was set at P of <0.05.

### Ethical approval

This study was conducted under the approval of the institutional review board (IRB) of Samsung Medical Center (IRB Number: 2020-11-143). All medical records at Samsung Medical Center were accessed from November 2020 to August 2021. As this study was a non-interventional retrospective study and all data were fully anonymized prior to access, the IRB waived the need for individual informed consent.

## Results

### Performance of CNN models

**Fig 5:**
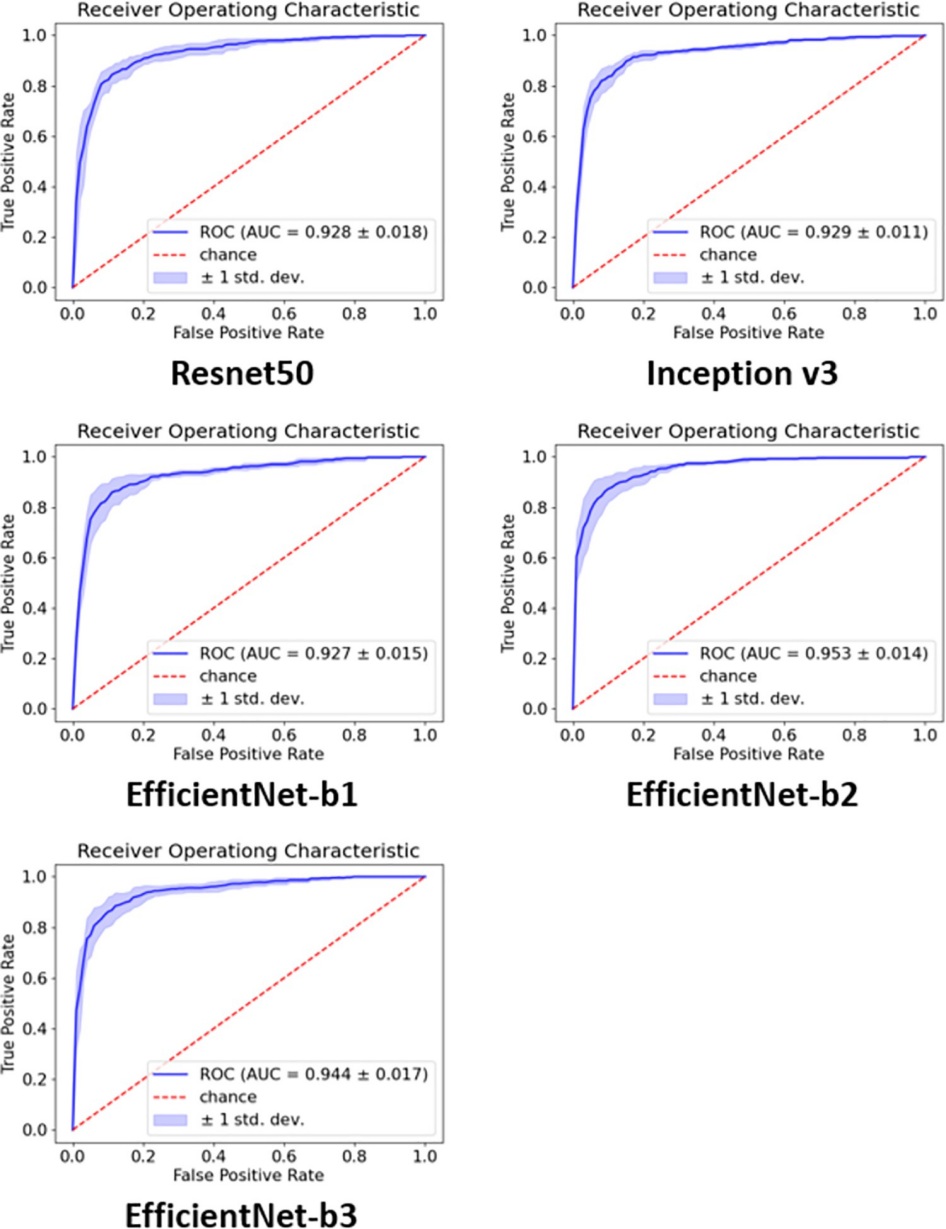
Micro ROC curves for each CNN model in the classification task. EfficientNet-b2 (0.953 AUC) outperformed the other networks. The mean and the standard deviation were given through 5-fold cross validation. ROC, receiver operating characteristic; CNN, convolutional neural network; AUC, area under the curve.

Table 1 lists the results of the three-label classification performed by each CNN model. Among the CNN models, EfficientNet-b2 outperformed the other models in all performance metrics. The mean accuracy, sensitivity, specificity, precision, and F1 score of the EfficientNet-b2 model were 0.853 ± 0.050, 0.822 ± 0.087, 0.912 ± 0.034, 0.829 ± 0.089, and 0.822 ± 0.065, respectively. The micro-average AUC for each CNN model is shown in Fig 5. The EfficientNet-b2 model outperformed the other CNN models in terms of AUC. EfficientNet-b2 had an AUC of 0.953 (95% CI, 0.926–0.980), whereas ResNet50 was 0.928 (95% CI, 0.893–0.963), Inception v3 was 0.929 (95% CI, 0.907–0.951), EfficientNet-b1 was 0.927 (95% CI, 0.898–0.956), and EfficientNet-b3 was 0.944 (95% CI, 0.911–0.977). As EfficientNet-b2 marked the best performance indicators, we adopted it as the representative evaluation model.

**Table 1:** Comparison of diagnostic performance for each Convolutional Neural Network (CNN) model.

CNN model	Accuracy	Sensitivity	Specificity	Precision	F1-Score
ResNet50	0.810 ± 0.046	0.764 ± 0.088	0.889 ± 0.046	0.780 ± 0.059	0.800 ± 0.045
Inception v3	0.821 ± 0.029	0.778 ± 0.087	0.892 ± 0.035	0.786 ± 0.054	0.778 ± 0.054
EfficientNet-b1	0.835 ± 0.054	0.784 ± 0.074	0.896 ± 0.033	0.809 ± 0.082	0.794 ± 0.067
EfficientNet-b2	**0.853 ± 0.050**	**0.822 ± 0.087**	**0.912 ± 0.034**	**0.829 ± 0.089**	**0.822 ± 0.065**
EfficientNet-b3	0.842 ± 0.040	0.787 ± 0.090	0.900 ± 0.036	0.818 ± 0.088	0.797 ± 0.068

The values are given as the mean and the standard deviation by 5-fold cross validation.

### Comparison with human doctors

The three-label classification performance was compared between the adopted CNN model (EfficientNet-b2) and four human doctors (Table 2). The diagnostic accuracy of the model (0.853) was significantly higher than the average value of four doctors (0.794) (P = 0.001) and was significantly higher than that of each human doctor (0.811, 0.796, 0.757, and 0.814, respectively) (P<0.05). The mean sensitivity, specificity, precision, and F1 score of the four doctors were 0.751 ± 0.069, 0.889 ± 0.028, 0.762 ± 0.070, and 0.797 ± 0.049, respectively. In particular, the adopted model outperformed the best scores from human doctors for all parameters, except for the F1 score from one doctor. Over 5% improvement in the accuracy, sensitivity, and precision was observed, when compared to the average of human doctors. In terms of specificity, the adopted model recorded a smaller performance improvement of approximately 2% as compared to the human average.

**Table 2:** Comparison of between the best performing Convolutional Neural Network (CNN) model and the human doctors.

	Accuracy	P-Value	Sensitivity	Specificity	Precision	F1 Score
**EfficientNet-b2**	**0.853 ± 0.050**		**0.822 ± 0.087**	**0.912 ± 0.034**	**0.829 ± 0.098**	**0.822 ± 0.065**
**Doctor 1**	0.811 ± 0.030	0.047	0.740 ± 0.079	0.887 ± 0.038	0.764 ± 0.074	0.791 ± 0.051
**Doctor 2**	0.796 ± 0.063	0.018	0.709 ± 0.105	0.888 ± 0.032	0.742 ± 0.107	0.768 ± 0.082
**Doctor 3**	0.757 ± 0.015	<0.001	0.819 ± 0.035	0.897 ± 0.019	0.768 ± 0.044	0.844 ± 0.025
**Doctor 4**	0.814 ± 0.032	0.004	0.736 ± 0.057	0.885 ± 0.022	0.775 ± 0.056	0.786 ± 0.039
**Mean Doctors**	**0.794 ± 0.035**	**0.001**	**0.751 ± 0.069**	**0.889 ± 0.028**	**0.762 ± 0.070**	**0.797 ± 0.049**

The values are given as the mean and the standard deviation. P-values were described to compare the diagnostic accuracy of the adopted CNN model (EfficientNet-b2) and that of the human doctors.

### Validity of image pre-processing

To verify the validity of image pre-processing procedures proposed in this study (Fig 2), the performance of the CNN model with and without applying these procedures were compared. In case of the model without the pre-processing procedures (i.e., basic scheme), the network was trained to perform the three-label classification (benign, malignant, or no tumor) using the original hip radiograph (i.e., the initial image in Fig 2). In such cases, all other procedures (e.g., cropping [Fig 3] and equalization procedures) were set in the same manner, except for the four pre-processing stages described in Fig 2. The size of all input images was adjusted to be 500×500. The best performing CNN algorithm (EfficientNet-b2) was used for comparison.

**Fig 6:**
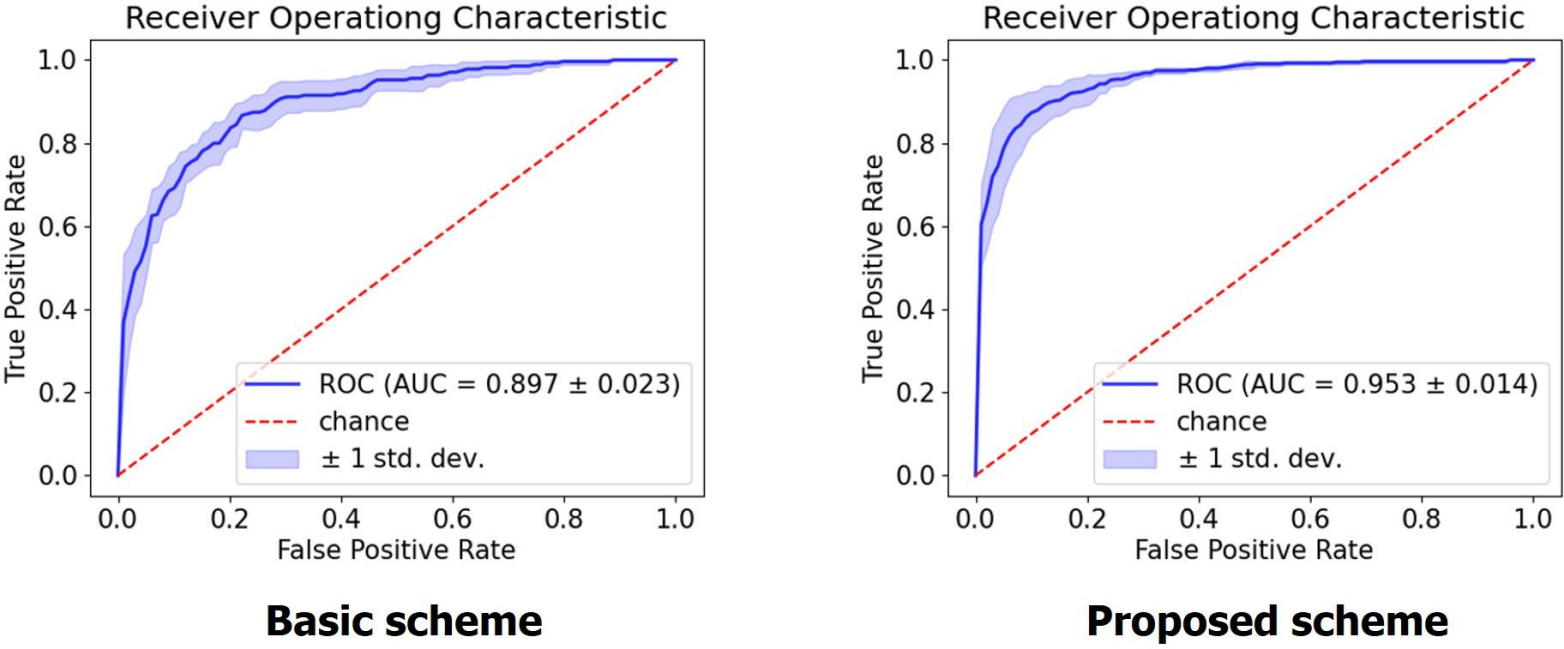
Micro ROC curves for EfficientNet-b2 model using the basic scheme and the proposed pre-processing method. The proposed method improved the AUC by more than 5%. The mean and the standard deviation were given through 5-fold cross validation. ROC, receiver operating characteristic; AUC, area under the curve.

The accuracy of the model with proposed pre-processing procedures (0.853 ± 0.050) was significantly higher than that of the basic scheme (0.747 ± 0.027) (Table 3). The average sensitivity (0.822 ± 0.087), specificity (0.912 ± 0.034), precision (0.829 ± 0.089), and F1-score (0.822 ± 0.065) of the model with pre-processing procedures were significantly higher than those of the basic scheme (0.738 ± 0.124, 0.878 ± 0.057, 0.735 ± 0.071, and 0.725 ± 0.070, respectively). The new pre-processing approach improved the mean sensitivity, specificity, precision, and F1-score by 0.084, 0.034, 0.094, and 0.104, respectively. ROC curves were plotted with the calculation of AUC (Fig 6). The AUC of the model with pre-processing (0.953; 95% CI, 0.926–0.980) was also higher than that of the basic scheme (0.897; 95% CI, 0.852–0.942).

**Table 3:** Comparison of diagnostic performance between using and not using the proposed pre-processing procedures.

	Accuracy	Sensitivity	Specificity	Precision	F1-Score
**Proposed scheme**	**0.853 ± 0.050**	**0.822 ± 0.087**	**0.912 ± 0.034**	**0.829 ± 0.089**	**0.822 ± 0.065**
**Basic scheme**	0.747 ± 0.027	0.738 ± 0.124	0.878 ± 0.057	0.735 ± 0.071	0.725 ± 0.070

The values are given as the mean and the standard deviation by 5-fold cross validation. The best performing CNN model (EfficientNet-b2) was used for comparison.

### Inference time for each model

The mean running time for each CNN model to diagnose bone tumors on both proximal femurs using individual hip radiograph is described in Table 4. All backbone CNN models produced a mean inference time of less than 0.1 seconds, supporting that the AI models can be effective for decision-making in the clinical setting.

**Table 4:** Inference time for each Convolutional Neural Network (CNN) model.

	Resnet50	Inception V3	EffcientNet-b1	EffcientNet-b2	EffcientNet-b3
**Inference time (seconds)**	**0.034** ± **0.014**	**0.049** ± **0.013**	**0.050** ± **0.014**	**0.048** ± **0.013**	**0.058** ± **0.015**

The values are given as the mean and standard deviation through validation of whole samples.

### Visualization of decision task

**Fig 7:**
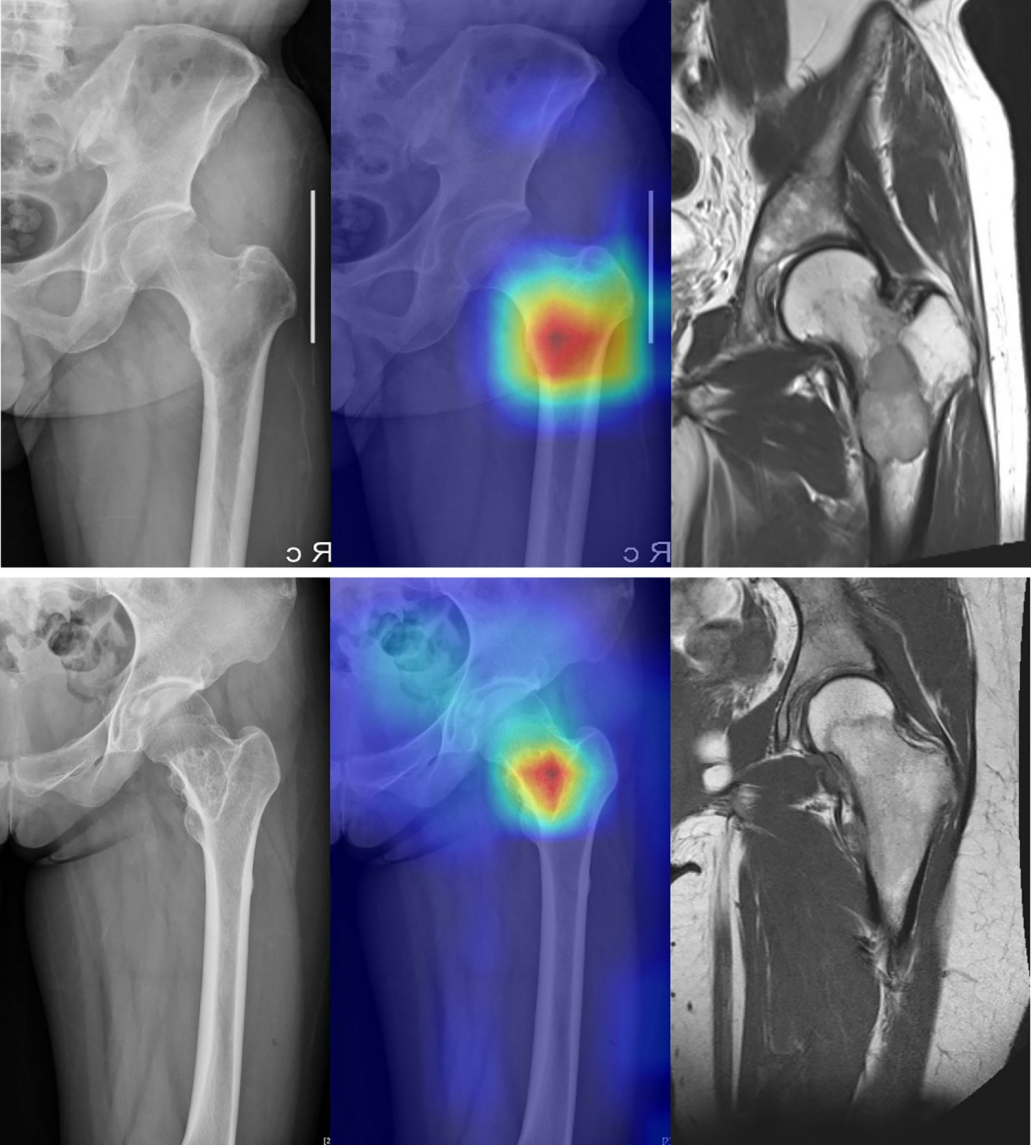
Gradient-weighted Class Activation Mapping (Grad-CAM) to show which part of the input image led to the classification decision of the deep learning model. The Grad-CAM revealed that the model classified the images based on the presence of tumors in the proximal part of each proximal femur. (a) Grad-CAM highlights the location of malignant tumor confirmed by magnetic resonance imaging (MRI). (b) Grad-CAM highlights the location of benign tumor confirmed by MRI.

To identify which part of the input image led to the final decision of the adopted CNN model, a technique called gradient-weighted class activation mapping (Grad-CAM) was used [11, 24]. Fig 7 shows the visualization results of Grad-CAM, which highlights the area between the head and trochanteric regions of each femur. In all images with benign and malignant tumors, Grad-CAM revealed that the model recognized tumors in the proximal femur.

## Discussion

In this study, we developed and validated an AI-based model to classify the presence of bone tumors in the proximal femur. We used five CNN algorithms to take 538 femoral images as training input to classify normal femurs and those with benign and malignant tumors. Among the five CNN models, EfficientNet-b2 model showed the best accuracy, sensitivity, specificity, precision, F1 score, and AUC. The diagnostic accuracy of EfficientNet-b2 model was significantly higher than that of the four human doctors Deep learning models with small parameters are known to be effective for learning small datasets [25]. Since EfficientNet-b2 requires fewer learning parameters than -b3, the learning data size in the current study was considered more suitable for -b2. Beyond the superiority of the AI model over human doctors shown in this study, future studies with more training data and larger networks would further advance the diagnostic performance of AI models in this subject.

Diagnosing bone tumors on plain radiographs is challenging, even for orthopaedic surgeons with considerable clinical experience or musculoskeletal radiologists. In general, human doctors diagnose bone tumors with “pattern recognition” using the location, shape, size, density, and margin of the tumor [8]. Among them, the most important clue is the margin, that is, the zone of transition [26]. A bone tumor with a narrow zone of transition, usually a benign tumor, is more apparent on radiographs. However, a malignant bone tumor is often not detected by human vision unless an obvious radiolucency or cortical destruction is observed. Although malignant bone tumors with osteoblastic or sclerotic characteristics can be more easily detected as they have a narrow zone of transition, they are often misdiagnosed as benign tumors [27]. Moreover, as the border of osteolytic tumors is unclear in patients with severe osteoporosis, the possibility of misdiagnosing them is high.

Significant progress has been made for AI-based analysis of plain radiographs [12, 13]. Characteristics used to diagnose bone tumors in radiographs, including the shape, matrix, density, and zone of transition, are considered suitable for application in the deep learning algorithm [8]. However, for musculoskeletal tumors, the application of machine learning technology has been sparsely reported. We found two reports that applied deep learning algorithms to detect bone tumors on plain radiographs. He et al. [8] applied a deep learning model to classify bone tumors on various radiographs from 1,356 patients. They obtained an AUC of 0.894 to distinguish between benign and non-benign tumors and an AUC of 0.907 to distinguish between malignant and non-malignant tumors. When the diagnostic accuracy of the model was compared with that of clinicians, it was higher than that of junior radiologists and similar to that of subspecialists. Do et al. [28] also developed a model to detect and classify bone tumors of the distal femur and proximal tibia and reported high diagnostic accuracy of 99%.

We found that the diagnostic performance of the CNN model was excellent in terms of accuracy, sensitivity, specificity, precision, and F1-score. One of the reasons for the high diagnostic accuracy was the use of standardized hip radiographs. Since anteroposterior hip radiographs can visualize lesions in the pelvic bone and both proximal femurs with low cost and effort, they are frequently used as the initial imaging test for patients complaining of hip and thigh discomfort. In addition, several image pre-processing stages have been adopted for hip radiographs to be better recognized by the AI model [15–18, 29]. The images were normalised using the min-max technique to reduce deviations between images due to variations in radiation exposure [15, 29]. Using the binarization process, the noise in the image was removed, and the boundary of the radiopaque area was clarified to identify the exact center of the body. Unnecessary black areas, letters, and markers in the image were removed through erosion and dilation [16].

The most important feature in the development of the current model is the division and flipping process to align the femoral images in one direction. Anteroposterior hip radiograph is one of the rare human radiographs that can be symmetrically divided into left and right. The flipping process, which converts all the right femur images to left femur images, not only helps the deep learning algorithm to focus more on a specific femoral lesion but also increases the number of normal femurs. The division process for radiographs with tumors on one side of the femur generates one femur with and one without the tumor, thereby increasing the normal data by approximately five times. This increase in the amount of normal data can further improve the classification performance of the model. As the division provides more information on normal data, the deep learning model can more clearly understand the difference between normal and abnormal cases.

An artificial neural network in deep learning algorithms is considered a black box. Recognizing what exact characteristics of the input data determine the decision of the algorithm is difficult. In practice, deep learning algorithms often find answers by learning from parts other than human interests. Grad-CAM is being used as a method to evaluate whether the AI is aware of the lesion of interest on the image [11, 12, 24]. We were able to confirm that the deep learning algorithm accurately recognizes the lesion of the proximal femur through Grad-CAM. Notably, we trained the CNN models without providing any information about the tumor location. From this point of view, the Grad-CAM also indicated that the CNN model learned the tumor location by itself, thereby verifying the validity of the high diagnostic performance of our model.

To our knowledge, the current study is the first to develop an AI model to classify bone tumors in proximal femurs on simple hip radiographs. One of the strengths of this classifier is that it is not a binary system that distinguishes normal femur and bone tumors, rather it is a tertiary output system that distinguishes normal tissues, benign tumors, and malignant tumors. We achieved high diagnostic performance owing to the pre-processing, which removed unnecessary information on images and increased the number of training images. This classifier is expected to have a high clinical utility because it uses a single radiograph commonly used in clinical practice.

This study has several limitations. First, the total number of hip radiographs used in this study was not sufficiently large. However, 214 femurs with bone tumors is not a small sample, considering the incidence of tumors occurring in the proximal femur. Second, patient information, such as age, sex, and clinical symptoms, was not used as an input for the deep learning ​​algorithm. Higher diagnostic performance can be expected when additional information is used in future models. Lastly, if the AI ​​model can provide information on the risk of pathological fractures in the proximal femur through the following study, the clinical utility of the model will be further increased.

## Conclusions

The AI-based model demonstrated excellent performance in classifying bone tumors in the proximal femur on plain hip radiographs and revealed a significantly higher diagnostic accuracy than that of practicing orthopaedic surgeons. Given the high accuracy and sensitivity of the diagnostic model developed in this study, AI-based technologies can potentially reduce the rate of misdiagnosis by doctors who are not specialists in musculoskeletal oncology.

## Supporting information

S1 Dataset Data used for analysis. (XLSX)
